# Short-term depression of inhibitory Purkinje cell synapses enhances gain modulation in the cerebellar nuclei

**DOI:** 10.1186/1471-2202-14-S1-P374

**Published:** 2013-07-08

**Authors:** Dimitris Bampasakis, Reinoud Maex, Neil Davey, Volker Steuber

**Affiliations:** 1 Science and Technology Research Institute, University of Hertfordshire, Hatfield AL10 9AB, UK; 2 Department of Cognitive Sciences, École Normale Supérieure, Paris 75005, France

## 

Information in neurons can be encoded by their action potential rate, thus making the transformation of input to output rate, the input-output (I-O) relationship, a core computational function. Introduction of a second input, often called modulatory input, can modify this I-O relationship in ways that correspond to different arithmetic operations [1]. Here, we examine the modulation of the slope of the I-O relationship, also referred to as gain modulation.

Gain modulation can be based on a wide variety of biophysical mechanisms, with short-term depression (STD) of excitatory synapses being one of them [2]. Commonly, gain modulation is studied by examining the effect of tonic or synaptic inhibition on the excitatory I-O relationship. However, some projection neurons, like cerebellar Purkinje cells (PCs), are inhibitory. Therefore, the opposite scenario, in which the effect of inhibition on output rate is being modulated by an excitatory input, may occur as well. As a previous study found that inhibitory synaptic input variability can change the output rate of neurons in the cerebellar nuclei (CN) [3], the question arises how excitatory input can modulate this relationship.

**Figure 1:**
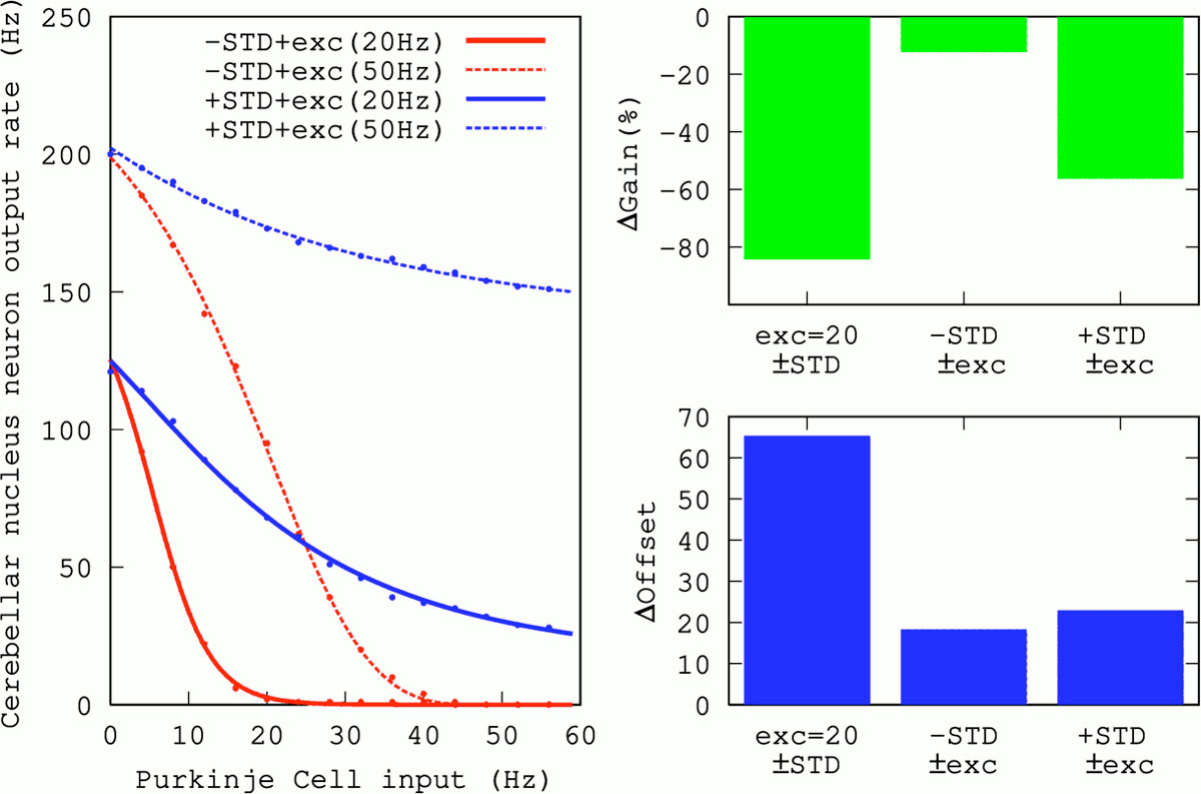
**Gain change due to excitatory modulatory input**. **Left: **Average output rate of the CN neuron as a function of PC inhibitory input, for 20 Hz and 50 Hz excitatory mossy fibre input. Dots and lines correspond to simulation data and fits of a Hill function, respectively. **Right: **Change in gain and offset (calculated as in [2]) in the presence and absence of STD for 20 Hz of excitatory rate (left bars), and for a change of the excitation from 20 Hz to 50 Hz in the absence (middle bars) and presence (right bars) of STD.

Considering the excitatory input from mossy fibres (MF) onto CN neurons as modulatory, we investigated the effects on gain control exerted by STD of the inhibitory synapses that PCs make on a model CN neuron [3]. We found that STD at the inhibitory PC-CN synapse enhanced gain modulation (Figure 1). Thus, like STD at excitatory synapses, STD at inhibitory synapses can enable neurons to perform multiplicative operations on their inputs.
